# Safety Profile of Warfarin and Apixaban for Deep Vein Thrombosis in Patients With Chronic Kidney Disease

**DOI:** 10.7759/cureus.98274

**Published:** 2025-12-01

**Authors:** Fatima Kausar, Osama Nadeem, Alina Khalil, Sajjad Ahmad, Wajid Ali

**Affiliations:** 1 Nephrology, Mayo Hospital, Lahore, PAK; 2 General Internal Medicine, North Manchester General Hospital, Manchester, GBR; 3 General Medicine, Mayo Hospital, Lahore, PAK; 4 Medicine, Shalamar Hospital, Lahore, PAK; 5 Internal Medicine, District Headquarters Hospital Timergara, Dir, PAK; 6 Surgery, Hayatabad Medical Complex Peshawar, Peshawar, PAK

**Keywords:** apixaban, bleeding events, chronic kidney disease (ckd), deep vein thrombosis (dvt), safety profile, warfarin

## Abstract

Background

Patients with chronic kidney disease (CKD) are more susceptible to bleeding complications and thromboembolic events. Current recommendations provide limited guidance on the optimal anticoagulant for CKD patients with deep vein thrombosis (DVT). Therefore, this study was planned. By estimating the proportion of bleeding episodes associated with apixaban and warfarin in CKD patients, this study aimed to address this knowledge gap and assist physicians in selecting safer and more effective treatment options.

Objective

The objective of this study is to compare the safety profiles of warfarin and apixaban for DVT in patients with CKD.

Methods

This descriptive comparative study was conducted at the Department of General Internal Medicine, Mayo Hospital, Lahore, Pakistan, from September 17, 2022, to September 16, 2024. Male and female patients aged 30 to 70 years with CKD, diagnosed with DVT, and started on warfarin or apixaban were enrolled. A total of 228 patients were included, with 114 in the apixaban group and 114 in the warfarin group. Patients were compared for the safety profiles of both drugs, assessed in terms of bleeding tendencies and worsening renal function. Data analysis was performed using IBM SPSS Statistics for Windows, Version 26.0 (Released 2018; IBM Corp., Armonk, NY, USA).

Results

The overall mean age of participants was 52 years. The mean age in the apixaban group was 54.73 ± 12.17 years, and in the warfarin group, it was 49.99 ± 12.14 years. Mean CKD duration in the apixaban and warfarin groups was 8.55 ± 2.14 years and 9.46 ± 2.73 years, respectively. Overall, 159 participants (69.7%) were male, with 80 (70.2%) in the apixaban group and 79 (69.3%) in the warfarin group. Participants over 50 years of age totaled 116 (50.9%), with 69 (60.5%) in the apixaban group and 47 (41.2%) in the warfarin group. Overall, 16 patients (7.0%) experienced a fatal bleed, including five (4.4%) in the apixaban group and 11 (9.6%) in the warfarin group. Major bleeding occurred in 16 patients (14.0%) receiving apixaban and 19 (16.7%) receiving warfarin. The chi-square p-values for differences in fatal bleed, major bleed, and minor or no bleed between apixaban and warfarin were 0.120, 0.582, and 0.153, respectively.

Conclusions

This study provided valuable information regarding the safety profile of warfarin and apixaban for DVT in individuals with CKD. The apixaban group experienced fewer fatal bleeding episodes than the warfarin group. Similarly, serious bleeding episodes were less frequent with apixaban compared with warfarin. However, there was no statistically significant difference between the two treatment groups. The study findings imply that apixaban might be a safer option than warfarin for anticoagulation in patients with CKD, but, due to the lack of statistical significance and the restriction of the study to a single center, further carefully designed prospective multicenter studies are warranted to validate these results.

## Introduction

Persistent renal insufficiency remains a leading cause of morbidity and mortality across nearly all age groups worldwide [1]. Chronic kidney disease (CKD) affects both developed and developing countries, significantly impacting healthcare expenditures [2]. Depending on the patient’s condition and the resources available, multiple treatment options exist for this chronic and debilitating illness. Even with treatment, prolonged renal failure can lead to multisystem complications that require an integrated approach from the healthcare team [3].

The various stages of CKD are associated with hematologic and metabolic derangements, which often worsen as the disease progresses [4]. Deep vein thrombosis (DVT) and atrial fibrillation are common consequences of these complications. Many patients experience these issues during the course of CKD and require anticoagulation therapy [5]. Before initiating anticoagulation and selecting a specific agent, healthcare providers must consider several factors [6].

Numerous studies have investigated the safety and efficacy of anticoagulants in patients with severe CKD, as these individuals may be predisposed to prothrombotic conditions [7,8]. In 2018, Chokesuwattanaskul et al. published a meta-analysis of five trials comparing warfarin and apixaban in patients with end-stage renal disease. The analysis found that apixaban was marginally safer and more effective than warfarin [9].

Safety profiles in these studies were primarily evaluated based on major bleeding events. In 2020, Hanni et al. assessed the effects of warfarin and apixaban in patients with CKD. Although apixaban was generally safer than warfarin, the overall adverse event profile showed little difference between the two drugs. Notably, apixaban was associated with fewer bleeding episodes compared with warfarin [10]. In another study of 135 patients with severe CKD treated for DVT, 55.6% were men and 44.4% were women. Of these, 66 patients (48.9%) received apixaban, and 69 patients (51.1%) received warfarin. Bleeding events occurred in 10.6% of patients taking apixaban and 21.7% of those taking warfarin [6].

Patients with CKD are more susceptible to bleeding complications and thromboembolic events due to comorbidities, impaired hemostasis, and altered drug metabolism. Because most studies excluded patients with severe renal impairment, current guidelines provide limited information on the optimal anticoagulant for CKD patients with DVT. By estimating the proportion of bleeding episodes associated with apixaban and warfarin in CKD patients, this study aims to address this knowledge gap and help physicians select safer and more effective treatment options. Improving patient outcomes and reducing anticoagulant-related complications in this vulnerable population could have a significant impact on clinical practice.

## Materials and methods

This descriptive comparative study was conducted at the Department of Internal Medicine, Mayo Hospital, Lahore, from September 17, 2022, to September 16, 2024, after approval from the institutional review board (approval no. 085/RC/KEMU). Male and female patients aged 20 to 80 years diagnosed with CKD and DVT were enrolled.

Exclusion criteria included a history of allergy to warfarin or apixaban, a history of bleeding diathesis, concurrent medications interfering with warfarin or apixaban, and failure to provide informed consent. CKD was defined using sonographic findings, including shrunken kidneys (size < 8 cm) with increased echogenicity and an estimated glomerular filtration rate of less than 60 mL/min/1.73 m². DVT was diagnosed sonographically by identifying a noncompressible venous segment with luminal echogenic foci and confirmed by Doppler imaging showing reduced or absent color flow in the deep veins of the limbs.

The safety profile was assessed based on bleeding events categorized as fatal (bleeding severe enough to cause death), major (hemoglobin drop > 2 g/dL from baseline requiring transfusion), minor (hemoglobin drop < 2 g/dL not requiring transfusion), and no bleeding events. All events were recorded within one month of initiating the medications. The study hypothesized that apixaban has a superior safety profile compared with warfarin in patients with CKD and DVT.

The sample size of 228 participants (114 per group) was calculated using OpenEpi software, assuming anticipated bleeding event rates of 10.6% for apixaban and 21.7% for warfarin, with 80% power and a 95% confidence level [6]. Participants were enrolled using a convenience sampling approach.

Informed consent was obtained from all participants after explaining the study purpose, procedures, risks, and benefits. Baseline demographic and clinical characteristics were recorded, including hemoglobin levels. Participants were randomly assigned to Group A (apixaban) or Group B (warfarin) using blocked randomization in equal numbers. Group A received apixaban 5 mg orally twice daily, while Group B received warfarin with doses adjusted to maintain an international normalized ratio of 2-3.

Patients were followed weekly for four weeks. Bleeding history was recorded, and hemoglobin levels were measured at baseline and at the final follow-up. Safety profiles were documented as fatal, major, or minor bleeding. Data were collected using a predesigned pro forma.

Data analysis was performed using IBM SPSS Statistics for Windows, Version 26.0 (Released 2018; IBM Corp., Armonk, NY, USA). Continuous variables were presented as mean ± standard deviation, while categorical variables were reported as frequencies and percentages. Bleeding events were compared between groups using the chi-square or Fisher’s exact test at a 5% significance level. Stratification was applied to control for potential effect modifiers, and post-stratification chi-square or Fisher’s exact tests were conducted. A p-value ≤ 0.05 was considered statistically significant.

## Results

The overall mean age of participants was 52.0 ± 12.36 years. The mean age was 54.73 ± 12.17 years in the apixaban group and 49.99 ± 12.14 years in the warfarin group. The overall mean BMI was 24.22 ± 1.01 kg/m², with 24.02 ± 1.06 kg/m² in the apixaban group and 24.42 ± 0.92 kg/m² in the warfarin group. The overall mean duration of CKD was 9.01 ± 2.49 years, with 8.55 ± 2.14 years in the apixaban group and 9.46 ± 2.73 years in the warfarin group.

Of the participants, 159 (69.7%) were male, including 80 (70.2%) in the apixaban group and 79 (69.3%) in the warfarin group. Participants over 50 years of age numbered 116 (50.9%), with 69 (60.5%) in the apixaban group and 47 (41.2%) in the warfarin group.

Overall, 47 patients (20.6%) had stage 5 CKD, including 20 (17.5%) receiving apixaban and 27 (23.7%) receiving warfarin. Dialysis was being received by 42 patients (36.8%) in the apixaban group compared with 40 patients (43.9%) in the warfarin group (Table 1).

**Table 1:** Baseline parameters of study participants CKD: chronic kidney disease; DM: diabetes mellitus; GN: glomerulonephropathies; HTN: hypertension

Parameter		Overall (n = 228)	Apixaban (n = 114)	Warfarin (n = 114)
Gender	Male	159 (69.7%)	80 (70.2%)	79 (69.3%)
	Female	69 (30.3%)	34 (29.8%)	35 (30.7%)
Age (years)	50 and below	112 (49.1%)	45 (39.5%)	67 (58.8%)
	Above 50	116 (50.9%)	69 (60.5%)	47 (41.2%)
BMI (kg/m²)	24.9 and below	172 (75.4%)	86 (75.4%)	86 (75.4%)
	Above 24.9	56 (24.6%)	28 (24.6%)	28 (24.6%)
CKD duration (years)	10 and below	163 (71.5%)	91 (79.8%)	72 (63.2%)
	Above 10	65 (28.5%)	23 (20.2%)	42 (36.8%)
CKD stage	Stage 3	56 (24.6%)	34 (29.8%)	22 (19.3%)
	Stage 4	125 (54.8%)	60 (52.6%)	65 (57.0%)
	Stage 5	47 (20.6%)	20 (17.5%)	27 (23.7%)
Treatment type	Conservative	136 (59.6%)	72 (63.2%)	64 (56.1%)
	Dialysis	92 (40.4%)	42 (36.8%)	50 (43.9%)
CKD etiology	DM	121 (53.1%)	54 (47.4%)	67 (58.8%)
	HTN	52 (22.8%)	31 (27.2%)	21 (18.4%)
	GN	31 (13.6%)	20 (17.5%)	11 (9.6%)
	Idiopathic	24 (10.5%)	9 (7.9%)	15 (13.2%)

Overall, 16 patients (7.0%) experienced a fatal bleed, including five patients (4.4%) in the apixaban group and 11 patients (9.6%) in the warfarin group. Major bleeding occurred in 16 patients (14.0%) receiving apixaban and 19 patients (16.7%) receiving warfarin (Table 2).

**Table 2:** Safety profile among the study participants (n = 228)

Safety profile	Overall (n = 228)	Apixaban (n = 114)	Warfarin (n = 114)
No/minor bleed	177 (77.6%)	93 (81.6%)	84 (73.7%)
Major bleed	35 (15.4%)	16 (14.0%)	19 (16.7%)
Fatal bleed	16 (7.0%)	5 (4.4%)	11 (9.6%)

The chi-square p-values for differences in the distribution of fatal bleed, major bleed, and minor or no bleed between the apixaban and warfarin groups were 0.120, 0.582, and 0.153, respectively (Table 3).

**Table 3:** Contingency table analysis of safety profile by drug administered (n = 228)

Safety profile		Group		Total	p-Value
		Apixaban (n = 114)	Warfarin (n = 114)		
Fatal bleed	Yes	5 (31.3%)	11 (68.8%)	16 (100.0%)	0.120
	No	109 (51.4%)	103 (48.6%)	212 (100.0%)	
Major bleed	Yes	16 (45.7%)	19 (54.3%)	35 (100.0%)	0.582
	No	98 (50.8%)	95 (49.2%)	193 (100.0%)	
Minor/no bleed	Yes	93 (52.5%)	84 (47.5%)	177 (100.0%)	0.153
	No	21 (41.2%)	30 (58.8%)	51 (100.0%)	

Subgroup analysis of fatal bleeding by age showed that eight patients (7.1%) were 50 years or younger, and eight patients (6.9%) were older than 50 years (p = 0.942). By gender, 11 male patients (6.9%) and five female patients (7.2%) experienced fatal bleeding (p = 0.929). No statistically significant associations were observed with other baseline parameters (p > 0.05) (Table 4).

**Table 4:** Subgroup analysis of fatal bleeding by baseline parameters CKD: chronic kidney disease; DM: diabetes mellitus; GN: glomerulonephropathies; HTN: hypertension

Parameter		Fatal bleed		Total	p-Value
		Yes (n = 16)	No (n = 212)		
Age (years)	50 or below	8 (7.1%)	104 (92.9%)	112 (100.0%)	0.942
	Above 50	8 (6.9%)	108 (93.1%)	116 (100.0%)	
Gender	Male	11 (6.9%)	148 (93.1%)	159 (100.0%)	0.929
	Female	5 (7.2%)	64 (92.8%)	69 (100.0%)	
BMI (kg/m²)	24.9 or below	13 (7.6%)	159 (92.4%)	172 (100.0%)	0.575
	Above 24.9	3 (5.4%)	53 (94.6%)	56 (100.0%)	
CKD stage	Stage 3	7 (12.5%)	49 (87.5%)	56 (100.0%)	0.106
	Stage 4	5 (4.0%)	120 (96.0%)	125 (100.0%)	
	Stage 5	4 (8.5%)	43 (91.5%)	47 (100.0%)	
Treatment type	Conservative	11 (8.1%)	125 (91.9%)	136 (100.0%)	0.442
	Dialysis	5 (5.4%)	87 (94.6%)	92 (100.0%)	
CKD etiology	DM	10 (8.3%)	111 (91.7%)	121 (100.0%)	0.324
	HTN	1 (1.9%)	51 (98.1%)	52 (100.0%)	
	GN	2 (6.5%)	29 (93.5%)	31 (100.0%)	
	Idiopathic	3 (12.5%)	21 (87.5%)	24 (100.0%)	
CKD duration (years)	10 or below	10 (6.1%)	153 (93.9%)	163 (100.0%)	0.409
	Above 10	6 (9.2%)	59 (90.8%)	65 (100.0%)	

Among patients with major bleeding, 28 (17.6%) were male, and seven (10.1%) were female (p = 0.151). Seventeen patients (12.5%) were receiving conservative treatment, compared to 18 patients (19.6%) receiving dialysis (p = 0.147) (Table 5).

**Table 5:** Subgroup analysis of major bleeding by baseline parameters CKD: chronic kidney disease; DM: diabetes mellitus; GN: glomerulonephropathies; HTN: hypertension

Parameter		Major bleed		Total	p-Value
		Yes (n = 35)	No (n = 193)		
Age (years)	50 or below	17 (15.2%)	95 (84.8%)	112 (100.0%)	0.943
	Above 50	18 (15.5%)	98 (84.5%)	116 (100.0%)	
Gender	Male	28 (17.6%)	131 (82.4%)	159 (100.0%)	0.151
	Female	7 (10.1%)	62 (89.9%	69 (100.0%)	
BMI (kg/m²)	24.9 or below	26 (15.1%)	146 (84.9%)	172 (100.0%)	0.863
	Above 24.9	9 (16.1%)	47 (83.9%)	56 (100.0%)	
CKD stage	Stage 3	10 (17.9%)	46 (82.1%)	56 (100.0%)	0.833
	Stage 4	18 (14.4%)	107 (85.6%)	125 (100.0%)	
	Stage 5	7 (14.9%)	40 (85.1%)	47 (100.0%)	
Treatment type	Conservative	17 (12.5%)	119 (87.5%)	136 (100.0%)	0.147
	Dialysis	18 (19.6%)	74 (80.4%)	92 (100.0%)	
CKD etiology	DM	17 (14.0%)	104 (86.0%)	121 (100.0%)	0.424
	HTN	9 (17.3%)	43 (82.7%)	52 (100.0%)	
	GN	3 (9.7%)	28 (90.3%)	31 (100.0%)	
	Idiopathic	6 (25.0%)	18 (75.0%)	24 (100.0%)	
CKD duration (years)	10 or below	28 (17.2%)	135 (82.8%)	163 (100.0%)	0.226
	Above 10	7 (10.8%)	58 (89.2%)	65 (100.0%)	

Among patients with no or minor bleeding, 102 patients (81.6%) had stage 4 CKD, 39 patients (69.6%) had stage 3, and 36 patients (76.6%) had stage 5 (p = 0.200). A total of 125 patients (76.7%) had a CKD duration of 10 years or less, while 52 patients (80.0%) had a duration of more than 10 years (p = 0.588) (Table 6).

**Table 6:** Subgroup analysis of no/minor bleeding by baseline parameters CKD: chronic kidney disease; DM: diabetes mellitus; GN: glomerulonephropathies; HTN: hypertension

Parameter		No/minor bleed		Total	p-Value
		Yes (n = 177)	No (n = 51)		
Age (years)	50 or below	87 (77.7%)	25 (22.3%)	112 (100.0%)	0.987
	Above 50	90 (77.6%)	26 (22.4%)	116 (100.0%)	
Gender	Male	120 (75.5%)	39 (24.5%)	159 (100.0%)	0.235
	Female	57 (82.6%)	12 (17.4%)	69 (100.0%)	
BMI (kg/m²)	24.9 or below	133 (77.3%)	39 (22.7%)	172 (100.0%)	0.846
	Above 24.9	44 (78.6%)	12 (21.4%)	56 (100.0%)	
CKD stage	Stage 3	39 (69.6%)	17 (30.4%)	56 (100.0%)	0.200
	Stage 4	102 (81.6%)	23 (18.4%)	125 (100.0%)	
	Stage 5	36 (76.6%)	11 (23.4%)	47 (100.0%)	
Treatment type	Conservative	108 (79.4%)	28 (20.6%)	136 (100.0%)	0.433
	Dialysis	69 (75.0%)	23 (25.0%)	92 (100.0%)	
CKD etiology	DM	94 (77.7%)	27 (22.3%)	121 (100.0%)	0.245
	HTN	42 (80.8%)	10 (19.2%)	52 (100.0%)	
	GN	26 (83.9%)	05 (16.1%)	31 (100.0%)	
	Idiopathic	15 (62.5%)	09 (37.5%)	24 (100.0%)	
CKD duration (years)	10 or below	125 (76.7%)	38 (23.3%)	163 (100.0%)	0.588
	Above 10	52 (80.0%)	13 (20.0%)	65 (100.0%)	

## Discussion

Our study found that individuals receiving apixaban experienced lower rates of fatal and major bleeding compared with those on warfarin; however, these differences were not statistically significant. Similarly, there was no statistically significant difference in the frequency of minor bleeding. Several supporting studies have evaluated the safety of apixaban in patients with kidney disease. Givens and colleagues examined bleeding rates in individuals using apixaban with and without renal disease. Although the frequency of major bleeding associated with apixaban was higher in individuals with renal disease than in those without, the differences in major and minor hemorrhage were not statistically significant (with renal disease, 7.8% versus without renal disease, 3.4%; p = 0.2) [11].

Apixaban demonstrated a more favorable bleeding profile than warfarin in patients with CKD, which may be explained by several factors. As established in prior research and landmark studies, apixaban, a direct factor Xa inhibitor, provides more consistent anticoagulant effects and substantially reduces the risk of severe bleeding [12]. Its short half-life allows rapid reversal of adverse effects, minimizing bleeding complications even if renal clearance is delayed. Additional factors that reduce bleeding risk include limited dietary interactions and predictable metabolism. Apixaban is also suitable for patients with mild to severe kidney dysfunction, as only 27% of the drug is renally eliminated. Collectively, these factors may account for the observed outcomes [13].

Subgroup analysis showed that individuals with advanced CKD had a higher risk of major bleeding and a greater need for blood transfusions; however, these differences were not statistically significant. Previous studies have similarly shown that patients with severe renal dysfunction are at increased risk of bleeding, regardless of anticoagulant therapy [14]. This indicates that the degree of renal impairment itself significantly elevates bleeding risk, underscoring the need for careful management of these patients. Additionally, patients with DVT have a higher likelihood of recurrent thromboembolic events compared with patients with atrial fibrillation. This highlights the importance of maintaining appropriate plasma levels of apixaban and associated anti-factor Xa activity [15].

The AMPLIFY study evaluated standard apixaban dosing for treating DVT in patients with adequate renal function (CrCl > 25 mL/min) [16]. Although FDA-approved labeling indicates that apixaban does not require renal dose adjustment for DVT regardless of CKD, it is important to note the limited number of clinical trials conducted in this population [17]. Given the higher baseline risk of recurrent venous thromboembolism (VTE) compared with atrial fibrillation, apixaban dosing must be carefully managed in DVT patients with renal impairment to prevent recurrent thromboembolic events while minimizing bleeding risk. In certain cases, such as renal failure, monitoring anti-Xa levels may be helpful, though routine surveillance is generally unnecessary [18].

Apixaban may be an appropriate alternative for patients with impaired or unstable kidney function unless there are strong indications for warfarin, such as a mechanical heart valve, mitral stenosis, or rheumatic heart disease. In patients with VTE and concomitant CKD, reducing the apixaban dose may increase the risk of recurrent thrombosis, thrombus propagation, or embolism [19].

To our knowledge, this study is the first to assess the real-world safety of apixaban compared with warfarin in patients with CKD. The analysis accounted for multiple potential confounders and included a sizable sample. However, several limitations must be acknowledged. First, the study was conducted at a single center, which may limit generalizability to other settings or populations. Second, the exact number of patients excluded during enrollment was not reported, introducing potential selection bias. Finally, the four-week follow-up period, used to assess short-term anticoagulation safety, limits the evaluation of long-term outcomes. Although this time frame allows assessment of early bleeding events, it cannot capture delayed complications.

## Conclusions

This study provides important insights into the safety profiles of apixaban and warfarin in patients with CKD. Apixaban was associated with a lower tendency for adverse events, including clinically significant bleeding, compared with warfarin. These findings suggest that apixaban may be a safer anticoagulant option for patients with CKD. However, as this study was conducted at a single center, the generalizability of the results may be limited. To confirm these findings and provide robust clinical evidence for treating DVT in CKD patients without exposing them to serious anticoagulation-related adverse events, carefully designed prospective multicenter studies with larger sample sizes are warranted.
